# Prior adaptation of parasitoids improves biological control of symbiont‐protected pests

**DOI:** 10.1111/eva.12934

**Published:** 2020-03-06

**Authors:** Silvan Rossbacher, Christoph Vorburger

**Affiliations:** ^1^ Aquatic Ecology Eawag Dübendorf Switzerland; ^2^ Institute of Integrative Biology ETH Zürich Zürich Switzerland

**Keywords:** aphids, biological control, defensive symbiosis, parasitoid, resistance evolution

## Abstract

There is increasing demand for sustainable pest management to reduce harmful effects of pesticides on the environment and human health. For pest aphids, biological control with parasitoid wasps provides a welcome alternative, particularly in greenhouses. However, aphids are frequently infected with the heritable bacterial endosymbiont *Hamiltonella defensa*, which increases resistance to parasitoids and thereby hampers biological control. Using the black bean aphid (*Aphis fabae*) and its main parasitoid *Lysiphlebus fabarum*, we tested whether prior adaptation of parasitoids can improve the control of symbiont‐protected pests. We had parasitoid lines adapted to two different strains of *H. defensa* by experimental evolution, as well as parasitoids evolved on *H. defensa*‐free aphids. We compared their ability to control caged aphid populations comprising 60% unprotected and 40% *H. defensa*‐protected aphids, with both *H. defensa* strains present in the populations. Parasitoids that were not adapted to *H. defensa* had virtually no effect on aphid population dynamics compared to parasitoid‐free controls, but one of the adapted lines and a mixture of both adapted lines controlled aphids successfully, strongly benefitting plant growth. Selection by parasitoids altered aphid population composition in a very specific manner. Aphid populations became dominated by *H. defensa*‐protected aphids in the presence of parasitoids, and each adapted parasitoid line selected for the *H. defensa* strain it was not adapted to. This study shows, for the first time, that prior adaptation of parasitoids improves biological control of symbiont‐protected pests, but the high specificity of parasitoid counter‐resistance may represent a challenge for its implementation.

## 1 INTRODUCTION

Agricultural intensification responds to the need of feeding a growing human population (Godfray et al., 2010). Intensive farming requires crop protection to limit yield losses to pest species. Chemical control with pesticides can be very effective, but it has devastating effects on biodiversity (Beketov, Kefford, Schafer, & Liess, 2013; Geiger et al., 2010) and it entails risks to human health (Alavanja, Hoppin, & Kamel, 2004; Kim, Kabir, & Jahan, 2017; Schwarzenbach, Egli, Hofstetter, Gunten, & Wehrli, 2010). Furthermore, rapid evolution of pest resistance limits the operational lifespan of pesticides (Hawkins, Bass, Dixon, & Neve, 2019). These concerns led to an increasing demand for sustainable alternatives, such as biological control with natural enemies (Heimpel & Mills, 2017).

Just like the application of insecticides, the introduction or the mass release of a natural enemy may impose strong selection on insect pests. Under the well‐supported assumption that insect populations harbor ample heritable variation for their susceptibility to natural enemies (Dubuffet et al., 2007; Henter & Via, 1995; Kraaijeveld & Godfray, 1997; Sandrock, Gouskov, & Vorburger, 2010), resistance to biocontrol agents is expected to evolve. However, Holt and Hochberg (1997) argued convincingly that the evolution of resistance to biological control is much less frequently observed than resistance to chemical pesticides. Possible reasons include temporally variable selection in the face of trade‐offs with resistance, weak selection as a consequence of spatial structuring, and importantly, coevolutionary dynamics (Holt & Hochberg, 1997). Parasitoids and predators exhibit genetic variation as well and are thus able to evolve counter‐resistance (Cavigliasso et al., 2019; Kraaijeveld, Hutcheson, Limentani, & Godfray, 2001), highlighting the opportunity for genetic improvement of biocontrol agents (Kruitwagen, Beukeboom, & Wertheim, 2018; Lommen, de Jong, & Pannebakker, 2017). Nevertheless, biological control is not immune to resistance evolution, as shown, for example, by the increasing resistance of Argentine stem weevils to the introduced parasitoid *Microctonus hyperodae* in New Zealand (Tomasetto, Tylianakis, Reale, Wratten, & Goldson, 2017).

Aphids are among the most important agricultural pests worldwide (Dedryver, Le Ralec, & Fabre, 2010). While chemical control of aphids still predominates in open fields, biological control of aphids has been adopted widely in greenhouse production, where the confined space facilitates the deployment of natural enemies (van Lenteren, 2012). An important component of the biocontrol arsenal against aphids is parasitoid wasps (Boivin, Hance, & Brodeur, 2012). Aphids may possess a particularly effective and intriguing defense in the form of heritable bacterial endosymbionts that have evolved the ability to protect their hosts against parasitoids (Oliver, Smith, & Russell, 2014; Vorburger, 2014). The best‐studied defensive symbiont of aphids is *Hamiltonella defensa* (Moran, Russell, Russell, Koga, & Fukatsu, 2005), which belongs to the Enterobacteriaceae and confers strong resistance to parasitoid wasps by killing the developing wasp larvae (Oliver, Russell, Moran, & Hunter, 2003; Schmid, Sieber, Zimmermann, & Vorburger, 2012). The ability to protect against parasitoids requires the presence of a toxin‐encoding bacteriophage called *Acyrthosiphon pisum* secondary endosymbiont (APSE) in *H. defensa's* genome (Brandt, Chevignon, Oliver, & Strand, 2017; Oliver, Degnan, Hunter, & Moran, 2009). The strength of protection provided by *H. defensa* is variable (Oliver, Moran, & Hunter, 2005) and exhibits considerable specificity, such that different strains are unequally effective against different parasitoid species (Asplen et al., 2014; Cayetano & Vorburger, 2015) or even different genotypes of the same species (Cayetano & Vorburger, 2013; Schmid et al., 2012). This variation may be related to the fact that different strains of *H. defensa* often carry variants of APSE that encode different primary toxins (Degnan & Moran, 2008; Moran, Degnan, Degnan, Santos, Dunbar, & Ochman, 2005).

Already when *H. defensa*‐conferred resistance to parasitoids was discovered, it was hypothesized that it may be responsible for observed failures of parasitoids to limit aphid abundance on crops (Gillespie, Quiring, Foottit, Foster, & Acheampong, 2009; Oliver et al., 2005). When tested in a laboratory setting, the introduction of parasitoids indeed resulted in a rapid increase of aphids harboring *H. defensa*, such that the increasingly resistant populations escaped control by parasitoids (Käch, Mathé‐Hubert, Dennis, & Vorburger, 2018). Defensive symbionts like *H. defensa* thus represent a challenge for biological control of pest aphids (Vorburger, 2018).

Unlike pesticides, biological control agents may evolve counter‐resistance, given genetic variation, including the resistance conferred by heritable endosymbionts. This was demonstrated by applying experimental evolution to *Aphidius ervi*, the main parasitoid of the pea aphid (*A. pisum*) (Dion, Zélé, Simon, & Outreman, 2011), and to *Lysiphlebus fabarum*, the main parasitoid of the black bean aphid (*Aphis fabae*) (Dennis, Patel, Oliver, & Vorburger, 2017; Rouchet & Vorburger, 2014). Both aphid species are important agricultural pests (Blackman & Eastop, 2017). Parasitoids adapted rapidly and showed a significantly improved ability to parasitize *H. defensa*‐protected aphids after only 4–10 generations of selection. In the case of *L. fabarum*, counter‐resistance was specific to the *H. defensa* strains carried by the aphids on which the wasps were evolved (Dennis et al., 2017; Rouchet & Vorburger, 2014). Such specificity is also a characteristic of natural populations of *L. fabarum*. A large collection of field‐collected lines varied widely in the ability to parasitize aphids infected with different strains of *H. defensa* (Vorburger & Rouchet, 2016).

These findings suggest that prior adaptation of parasitoids to aphids carrying protective symbionts could be a viable strategy to improve biological control of pest aphids in which such symbionts occur. We tested this hypothesis by deploying experimentally evolved lines of parasitoids from previous work (Dennis et al., 2017) in caged populations of black bean aphids. We found that prior adaptation to *H. defensa* can indeed allow parasitoids to control aphid populations that would otherwise escape control due to the rapid evolution of symbiont‐conferred resistance. This result represents a proof of principle for the genetic improvement of biocontrol agents by experimental evolution.

## 2 MATERIALS AND METHODS

### 2.1 Insect lines

As hosts, we used one *H. defensa*‐free and two *H. defensa*‐infected clonal lines of *A. fabae* with the same genetic background. The common genetic background was a single clone of *A. fabae* referred to as A06–407. It was collected in July 2006 from *Chenopodium album* in St. Margrethen (Switzerland) and does not contain any known facultative endosymbionts of aphids (Vorburger, Sandrock, Gouskov, Castañeda, & Ferrari, 2009). The two *H. defensa*‐positive lines were generated by microinjection of *H. defensa*‐containing hemolymph from two different donor clones (A06‐76 and A06‐402) into clone A06‐407, which resulted in stable, heritable infections with *H. defensa* (Rouchet & Vorburger, 2014). The infected lines are designated as A06‐407^H76^ and A06‐407^H402^, where H76 and H402 refer to the two strains of *H. defensa*. These strains are clearly distinct based on sequences of two housekeeping genes, and they contain different variants of the APSE phage encoding different toxins (Dennis et al., 2017).

As parasitoids, we used three sexual stocks of *L. fabarum* that have a common origin but differ in their history of laboratory adaptation to aphid hosts. These populations are derived from an experimental evolution study described in Dennis et al. (2017). Briefly, four replicate populations of *L. fabarum* were reared on each of the three aphid lines described above: A06‐407, A06‐407^H76^, and A06‐407^H402^, which resulted in significant and specific counteradaptation of parasitoids to *H. defensa*‐conferred resistance. Parasitoids reared on each of the *H. defensa*‐infected lines evolved increased infectivity (ability to overcome host resistance and successfully parasitize hosts) on aphids possessing their but not the other strain of *H. defensa*, whereas aphids reared on *H. defensa*‐free aphids remained poorly infective on either of the *H. defensa*‐protected lines (Dennis et al., 2017). Parasitoid adaptation to *H. defensa*‐protected aphids did not entail any obvious correlated responses in terms of parasitoid life‐history traits or a reduced ability to parasitize unprotected aphids. The experiment was terminated after 24 generations by merging the four replicate populations of the same treatment into a single population. The three evolved populations are still maintained on their respective hosts in our laboratory. By the start of the present experiment, they had been reared on these aphid lines for *c*. 120 generations, and they had maintained the described pattern of specific adaptation (data not shown).

### 2.2 Experimental procedures

Aphid populations were reared in 25 polyester insect cages with dimension 32.5 × 32.5 × 32.5 cm (BugDorm‐4F3030; MegaView Science). Cages initially contained four potted broad bean plants (*Vicia faba*, aged 14 days) and were placed on a single shelf in a climatized room set at 22°C with a 16‐hr photoperiod. Under these conditions, *A. fabae* reproduces by viviparous parthenogenesis exclusively. Aphid populations were started by adding 15 adult females of line A06‐407 and 5 adult females each of A06‐407^H76^ and A06‐407^H402^. Forty percent infection with *H. defensa* corresponds closely to the prevalence of this symbiont in the natural populations we study (Vorburger et al., 2009). The addition of aphids marked day 0 of the experiment. Four days later, two additional plants were added to the cages and cages were assigned randomly to one of five treatments (5 replicates each): control (no parasitoids); H‐ (parasitoids evolved on *H. defensa*‐free aphids); H76 (parasitoids evolved on H76‐infected aphids); H402 (parasitoids evolved on H402‐infected aphids); and H76 + H402 (mixture of parasitoids evolved on H76‐ and on H402‐infected aphids). Treatments were applied on day 7 of the experiment by adding 22 female and 12 male wasps of the respective lines of *L. fabarum* to each cage (11 and 6 each for the mixed treatment). On the same day, aphid density was estimated for the first time; thereafter, aphid and parasitoid density was estimated twice per week. On each occasion, we removed the two oldest plants from the cages and replaced them with two new plants. One of the removed plants was selected randomly for counting, the other was cut and returned to the cage so that aphids could migrate over to live plants and parasitoid mummies on the plants could hatch inside the cage. The retained plant was sealed in a plastic bag and frozen to arrest aphid reproduction, before all aphids and parasitoid mummies were counted. After day 18 of the experiment, plants started to show stunted growth because of aphid infestations; hence, we began to quantify plant size. For this, the plants were disassembled into stalks and leaves, which were spread out and photographed on a white background with size reference. The area of all plant material in the photographs was estimated with ImageJ v. 1.52 (Schneider, Rasband, & Eliceiri, 2012), from which we calculated the variable “plant surface” = 4 × total stalk area + 1 × total leaf area, since aphids feed on the underside of leaves and on the stalks, which have a quadratic cross section in *V. faba*. The last count took place 67 days after the addition of the aphids to the cages. At this point, we also took a haphazard sample of 24 aphids per cage to quantify their population composition at the end of the experiment, and we determined the total fresh weight of all plants in the cages (aboveground parts) as a measure of plant condition.

### 2.3 Final composition of aphid populations

The DNA of aphids collected at the end of the experiment was extracted using the “salting out” protocol described in (Sunnucks and Hales, 1996). We tested each individual for infection with *H. defensa* by diagnostic PCR, amplifying part of the 16S ribosomal RNA gene with symbiont‐specific primers (Ferrari, West, Via, & Godfray, 2012). We also ran a diagnostic PCR for the obligate endosymbiont of aphids, *Buchnera aphidicola*, as a control to verify the presence of amplifiable symbiont DNA in the extractions. For all *H. defensa*‐positive individuals, we identified the *H. defensa* strain by amplifying and Sanger sequencing a fragment of the bacterial housekeeping gene Murein (*murE*) as in Cayetano, Rothacher, Simon, and Vorburger (2015). It turned out that the diagnostic PCR for *H. defensa* was somewhat more sensitive than that for *B. aphidicola*, because for a few DNA extractions from very small aphids, we obtained a clear amplification product for *H. defensa* but not for *B. aphidicola*. To avoid any bias, the proportion of *H. defensa*‐positive and *H. defensa*‐negative aphids per cage was estimated only from samples that tested positive for *B. aphidicola*, but the relative frequencies of infection with H76 or H402 among the *H. defensa*‐positive aphids were estimated from all samples for which a *murE* sequence could be obtained.

### 2.4 Statistical analyses

Aphid density on plants was expressed as individuals per cm^2^, that is, #aphids/plant surface area, and parasitoid density as the number of mummies per cm^2^. To obtain comparable values from the early counts when plant growth was not visibly impacted by aphids and plant size not quantified, we assumed a plant surface of 149.8 cm^2^, which is the average of healthy plants of the same age. Densities were analyzed with mixed linear models after square root transformation for aphid densities and log‐transformation for parasitoid densities (log(#mummies + 1)/plant surface area) to improve normality of residuals and homogeneity of variances. We tested for the effects of treatment, time (day of count), and the treatment × time interaction. Cage was included as a random effect to account for the nonindependence of successive counts from the same cage. For aphid and parasitoid densities, we ran global models with all treatments as well as models for all pairwise comparisons between treatments with sequential Bonferroni correction to account for multiple testing (Rice, 1989). Analyses were carried out in R v. 3.5.0 (R Core Team, 2017), using the *lme4* library (Bates, Maechler, Bolker, & Walker, 2015) with the *lmerTest* library for significance tests of fixed and random effects (Kuznetsova, Brockhoff, & Christensen, 2015).

Plant fresh weights at the end of the experiment were compared among treatments with a one‐way ANOVA, followed by pairwise comparisons using Tukey's HSD (Tukey, 1977). The final composition of aphid populations was analyzed with permutational MANOVA on the arcsine square root‐transformed proportions of *H. defensa*‐free, H76‐infected, and H402‐infected aphids, using the *adonis* function in the *vegan* library (Oksanen et al., 2019), followed by pairwise post hoc comparisons with the *pairwise.perm.manova* function in the *RVAideMemoire* package (Hervé, 2020). For all treatments, we also tested whether the total proportion of *H. defensa*‐infected aphids at the end of the experiment differed from the initial proportion of 0.4, using one‐sample *t* tests.

## 3 RESULTS

**Figure 1:**
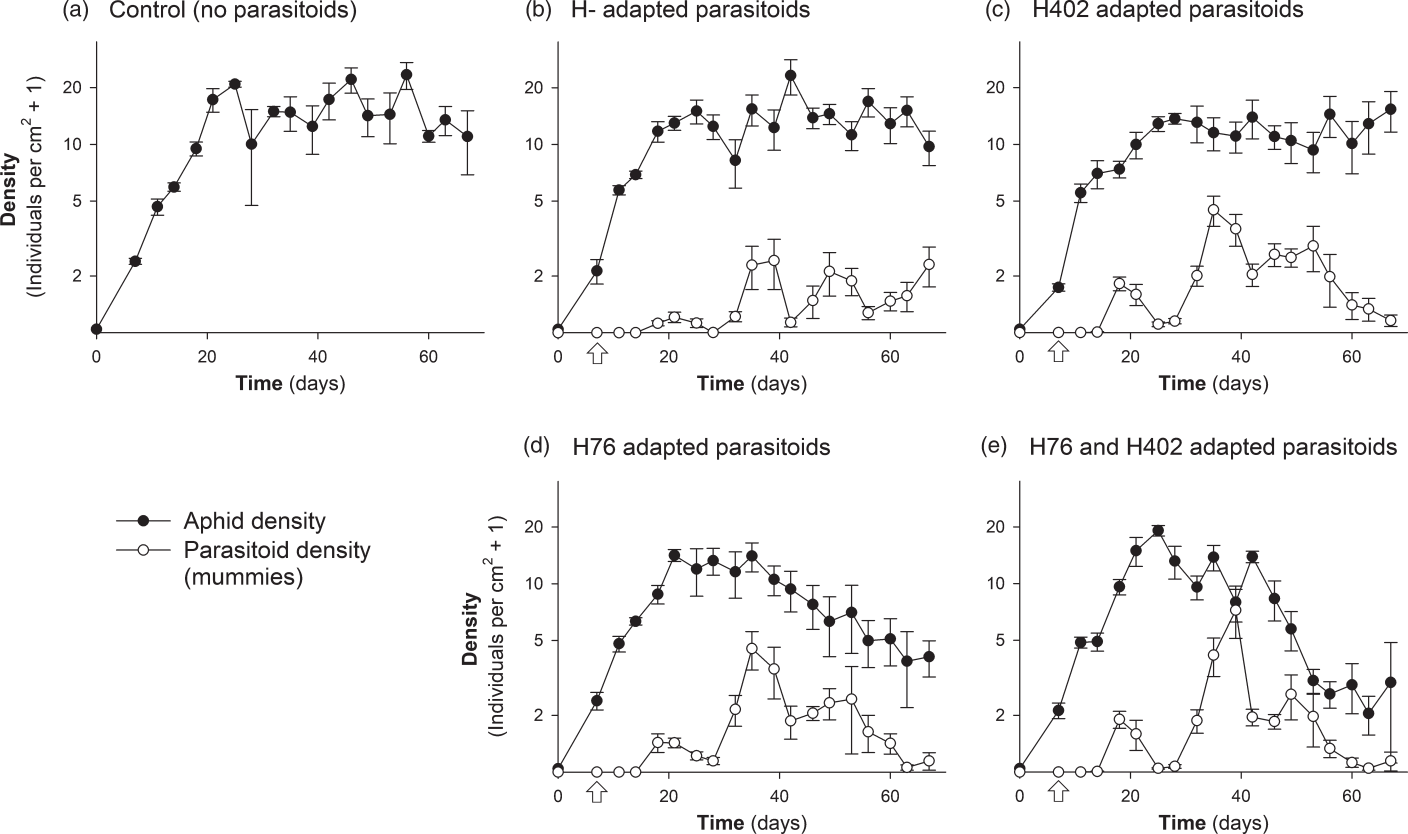
Dynamics of aphid and parasitoid population densities in the five experimental treatments. Values depict means of five replicate cages ± 1 *SE*. Arrows indicate the introduction of parasitoids to the cages. Note that the *y*‐axis is on a logarithmic scale

The treatments had a clear effect on the aphid population dynamics (LMM, treatment: *F*_4,20_ = 10.992, *p* < .001; time: *F*_18,360_ = 33.656, *p* < .001; treatment × time: *F*_72,360_ = 2.893, *p* < .001), and also, the parasitoid population dynamics differed significantly among the four treatments that contained wasps (treatment: *F*_3,16_ = 1.566, *p* = .237; time: *F*_18,288_ = 67.088, *p* < .001; treatment × time: *F*_54,288_ = 2.655, *p* < .001) (Figure 1). Specifically, when we introduced *L. fabarum* from a population that was evolved experimentally on *H. defensa*‐free (H‐) aphids, the parasitoids established successfully but had no detectable effect on aphid population density compared to parasitoid‐free control cages (Figure 1a, b, Table 1). When we introduced parasitoids from a population that was evolved on H402‐infected aphids (i.e., parasitoids adapted to aphids carrying *H. defensa* strain H402), parasitoids reached higher densities than the parasitoids adapted to H‐ aphids, but the effect on aphid populations remained weak, such that aphid densities, although somewhat lower, did not differ significantly from the control and the H‐ treatments (Figure 1c, Tables 1 and 2). However, parasitoids adapted to H76‐infected aphids did have a significant effect on aphid population dynamics. They suppressed aphid population growth, resulting in significantly lower aphid densities at the end of the experiment (Figure 1d, Table 1). Virtually the same result was observed when we introduced a mixture of H76‐ and H402‐adapted parasitoids (Figure 1e, Table 1).

**Table 1:** Tests of the treatment effect and the treatment × time interaction on aphid densities for all pairwise comparisons between treatments

Comparison	Treatment		Treatment × Time	
	*F*_1,8_	*p*	*F*_18,144_	*p*
Control versus H‐	0.395	.547	1.110	.349
Control versus H402	3.028	.120	1.422	.129
Control versus H76	14.995	**.005**	3.415	**<.001**
Control versus H76 + H402	44.350	**<.001**	5.566	**<.001**
H‐ versus H402	2.057	.189	0.990	.474
H‐ versus H76	14.578	**.005**	3.152	**<.001**
H‐ versus H76 + H402	64.898	**<.001**	5.723	**<.001**
H402 versus H76	4.126	.077	2.289	**.004**
H402 versus H76 + H402	11.852	.009	5.280	**<.001**
H76 versus H76 + H402	0.360	.565	1.116	.342

Note

*p*‐Values in bold print are significant after sequential Bonferroni correction for a table‐wide *α* = 0.05.

**Table 2:** Tests of the treatment effect and the treatment × time interaction on parasitoid densities for all pairwise comparisons between treatments that contained parasitoids

Comparison	Treatment		Treatment × Time	
	*F*_1,8_	*p*	*F*_18,144_	*p*
H‐ versus H402	3.030	.120	4.187	**<.001**
H‐ versus H76	0.711	.424	4.661	**<.001**
H‐ versus H76 + H402	0.895	.372	7.310	**<.001**
H402 versus H76	1.618	.239	0.549	.929
H402 versus H76 + H402	2.517	.151	0.603	.893
H76 versus H76 + H402	0.000	.992	0.739	.766

Note

*p*‐Values in bold print are significant after sequential Bonferroni correction for a table‐wide *α* = 0.05.

**Figure 2:**
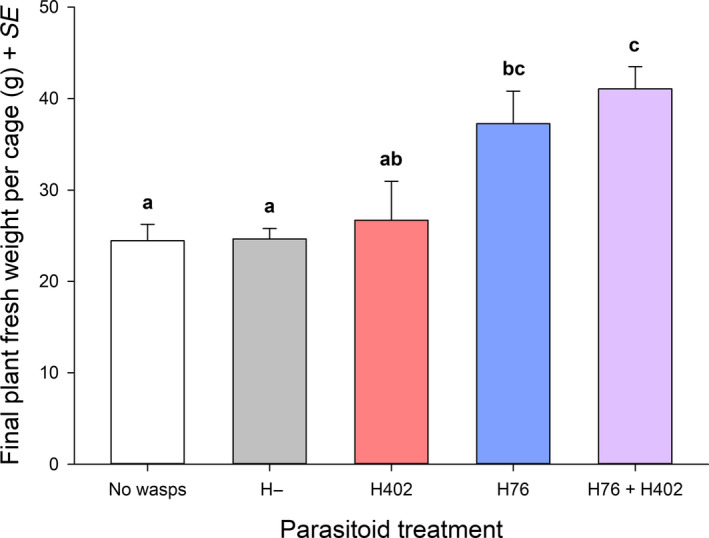
Total plant fresh weights per cage differed significantly among treatments at the end of the experiment (ANOVA, *F*_4,20_ = 7.351, *p* < .001). Treatments with different letters are significantly different in pairwise post hoc tests (Tukey's HSD). Error bars depict 1 *SE*

The broad bean plants clearly benefitted from successful aphid control in that plant fresh weights at the end of the experiment were highest in the two treatments where parasitoids managed to suppress aphid populations (Figure 2).

**Figure 3:**
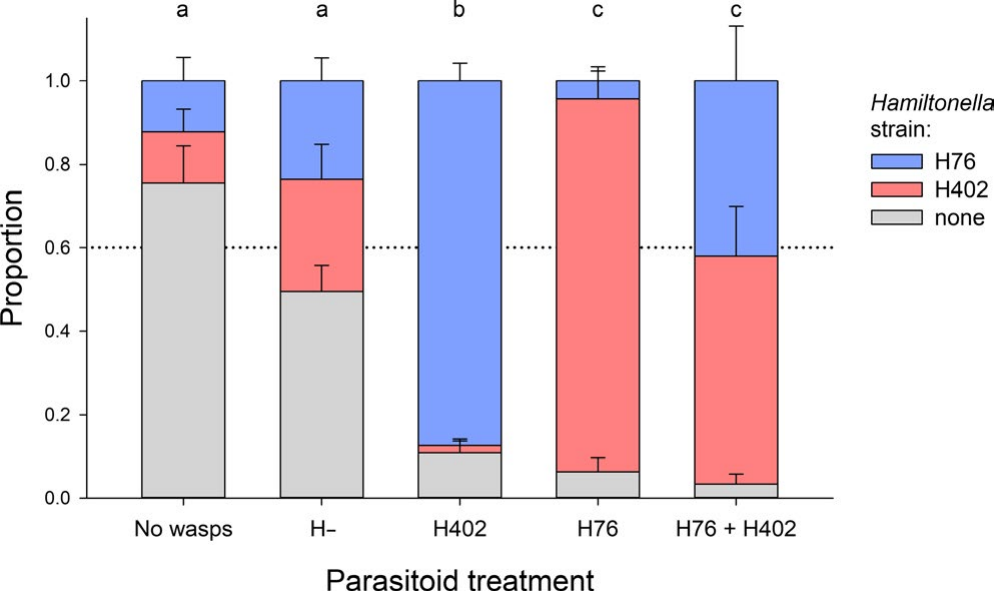
The composition of aphid populations differed significantly among treatments at the end of the experiment (permutational MANOVA, *F*_4,20_ = 20.30, *p* < .001). Treatments with different letters are significantly different in pairwise post hoc tests. Error bars depict 1 *SE*. The dashed line indicates the proportion of *H. defensa*‐free aphids at the beginning of the experiment

The aphid population compositions differed significantly among treatments at the end of the experiment (permutational MANOVA, *F*_4,20_ = 20.30, *p* < .001) and reflected the specificity of the selection imposed by the differently adapted parasitoids (Figure 3). All treatments differed significantly from each other in pairwise post hoc comparisons except for control versus H‐ and H76 versus H76 + H402 (Figure 3). In the control cages without parasitoids, the proportion of *H. defensa*‐infected aphids had declined somewhat by the end of the experiment, likely reflecting the known cost of harboring *H. defensa* in the absence of parasitoids (Oliver, Campos, Moran, & Hunter, 2008; Vorburger & Gouskov, 2011), although this decline was not significant (*t*_4_ = −1.746, *p* = .156). All other treatments showed a weak (H‐ treatment: *t*_4_ = 1.677, *p* = .169) or strong (H76, H402, H76 + H402: all *p* < .001) increase of aphids possessing *H. defensa*. When the parasitoids were adapted to H402‐infected aphids, the H402 strain nearly disappeared from the cages and the *H. defensa*‐positive aphids mostly carried H76, whereas the opposite was the case when H76‐adapted parasitoids had been added to the cages. The proportions of the two *H. defensa* strains remained more balanced when the parasitoids comprised H76‐ and H402‐adapted wasps (Figure 3).

## 4 DISCUSSION

There is an urgent need for more sustainable pest control to reduce the harmful side effects of conventional control with pesticides (Geiger et al., 2010; Kim et al., 2017). Biological control with parasitoids is a much‐used alternative to control pest aphids, particularly in greenhouse crops (Boivin et al., 2012), but its success can be hampered by the rapid evolution of symbiont‐conferred resistance (Käch et al., 2018). Here, we show that parasitoids with improved infectivity through prior adaptation to defensive symbionts present in an aphid pest can reduce aphid population densities in a situation where unselected parasitoids fail. Improving biological control agents through selective breeding is not a new approach (Kruitwagen et al., 2018; Lommen et al., 2017). It has been applied to life‐history traits such as development time and sex ratio, to behavioral traits like host finding, and to increased tolerance of host defenses (reviewed in Lirakis & Magalhães, 2019). We show for the first time, though, that parasitoid adaptation to a defense encoded by a microbial symbiont rather than the host itself does indeed improve control of a symbiont‐protected pest. Aphid population suppression resulted in improved plant growth, which is the main goal of biological control.

A potential problem of this approach is the high specificity of resistance conferred by *H. defensa* and the counteradaptations of parasitoids, because multiple strains of this symbiont with different defensive properties may occur in the same aphid species (Cayetano et al., 2015; Henry et al., 2013; Oliver & Higashi, 2019). With the two *H. defensa* strains used here, there is virtually no cross‐infectivity of the experimentally evolved parasitoids. Although they attack them normally, parasitoids are unable to develop in aphids infected with the alternative strain of *H. defensa* (Dennis et al., 2017). Cross‐infectivity may evolve, however, when the selection regimes include more similar strains of *H. defensa* (Rouchet & Vorburger, 2014).

The specific counter‐resistance of experimentally evolved parasitoids was also reflected in how the aphid populations responded to selection by parasitoids. When H402‐adapted parasitoids were applied, aphid populations became dominated by H76‐infected aphids, and when H76‐adapted parasitoids were applied, almost only H402‐infected aphids survived. In the first case, this rapid response to selection prevented an effective control of aphid populations (Figure 1c), but in the second case, the parasitoids were still able to suppress aphid densities (Figure 1d). This difference is interesting, albeit not entirely explicable by our current knowledge of the system. We can exclude that H76‐adapted wasps also parasitized H402‐infected aphids effectively, because tests immediately before and after the present cage experiment confirmed that there was no cross‐infectivity, as reported in Dennis et al. (2017) (data not shown). However, when the presence of a susceptible host population supports sufficiently high parasitoid densities, wasps can also kill resistant aphids, not through parasitism but presumably by continuous disturbance that prevents aphid feeding and leads to starvation, or by excessive stabbing (Hertäg & Vorburger, 2018). These would be examples of nonreproductive effects of insect parasitoids on their hosts (Abram, Brodeur, Urbaneja, & Tena, 2019). H76‐adapted wasps possibly affected H402‐protected aphids more strongly via such mechanisms than *vice versa*. It is also feasible that the difference in aphid control between the H76 and H402 treatments (Figure 1c, d) was a consequence of different cost–benefit ratios of the two *H. defensa* strains for their aphid hosts. Although beneficial against parasitoids, infection with *H. defensa* also entails costs for *A. fabae* in terms of reduced lifespan and lifetime reproduction (Vorburger & Gouskov, 2011). This could explain the slight decline of *H. defensa*‐infected aphids in the wasp‐free treatment. In a comparison of multiple *H. defensa* strains, it has been shown that H402 is more costly to the host than H76 (Cayetano et al., 2015). Thus, we could speculate that the H76‐adapted wasps have exerted better control because they selected for H402‐infected aphids that are generally less fit than the H76‐infected aphids that were favored by the H402‐adapted parasitoids. Whatever the correct explanation, using a combination of parasitoids adapted to different symbiont strains, as we did in one of our treatments, may safeguard against this issue (Figure 1e). An alternative approach would be to expose parasitoids to selection regimes comprising more than one strain of *H. defensa*, either simultaneously or sequentially, in the hope to produce more general counteradaptation that is effective against multiple strains of *H. defensa*. To our knowledge, this has never been tried. It is thus unknown whether or not such an approach would be foiled by the strong specificity of *H. defensa*‐conferred resistance (Cayetano & Vorburger, 2013; Schmid et al., 2012; Vorburger & Rouchet, 2016).

How does this specificity come about? Our working hypothesis is that specificity is a consequence of the underlying mechanism of *H. defensa*‐conferred resistance. It has been shown that different strains of *H. defensa* possess variants of the APSE phage that encode different toxins (Degnan & Moran, 2008; Moran, Degnan, et al., 2005), which also applies to the two strains used here. The APSE variant associated with *H. defensa* strain H76 encodes a 1,587 amino acid YD‐repeat protein as a putative toxin (NCBI GenBank: KU175898), whereas *H. defensa* strain H402 contains an APSE variant encoding a 293 amino acid homolog of cytolethal distending toxin (CdtB, NCBI GenBank: KU175897) (Dennis et al., 2017). These toxins are likely to represent different challenges for the parasitoids that need to be overcome by different counteradaptations. To determine whether this is indeed the case requires additional work, which will be facilitated by the recent development of culture‐based methods to study *H. defensa* (Brandt et al., 2017; Chevignon, Boyd, Brandt, Oliver, & Strand, 2018).

Symbiont‐conferred resistance to parasitoids is not restricted to aphids. Since the original discovery in pea aphids (Oliver et al., 2003), new cases keep being discovered (Hansen, Jeong, Paine, & Stouthamer, 2007; Xie, Butler, Sanchez, & Mateos, 2014; Xie, Vilchez, & Mateos, 2010). Protection by heritable endosymbionts may also extend to other parasites and pathogens of current or potential use in biological control. Examples include the protection by several endosymbiont species against entomopathogenic fungi in aphids (Łukasik, Asch, Guo, Ferrari, & Godfray, 2013; Scarborough, Ferrari, & Godfray, 2005), or the *Wolbachia*‐mediated protection against viral pathogens in flies and mosquitoes (Glaser & Meola, 2010; Hedges, Brownlie, O'Neill, & Johnson, 2008; Teixeira, Ferreira, & Ashburner, 2008). Thus, we expect that future research will show that defensive symbionts can challenge the biological control of various arthropod pests. Just as in pesticides, strong selection by biological control agents will favor the evolution of resistance (Tomasetto et al., 2017). In pest populations where defensive symbionts occur, this will result in an elevated prevalence of these symbionts that may reduce biocontrol success (Käch et al., 2018; Oliver et al., 2008). Unlike pesticides, however, biological control agents possess genetic variation to evolve counter‐resistance. This genetic variation can be managed and selected to improve the performance of natural enemies for biological control (Kruitwagen et al., 2018; Lommen et al., 2017). Here, we provided a proof of principle, in a laboratory setting, that experimental evolution is an effective means to improve the biocontrol capacity of parasitoid wasps toward symbiont‐protected pests. The confined space and the simplified single‐crop habitat typical of greenhouse cultures certainly bear similarity to a laboratory setting, but it remains to be demonstrated whether the approach can also be applied successfully at the larger scale of real biocontrol interventions.

## CONFLICT OF INTEREST

None declared.

## Data Availability

Data are available at Dryad Digital Repository: https://doi.org/10.5061/dryad.tht76hdw4.
